# Galectins as Cancer Biomarkers

**DOI:** 10.3390/cancers2020592

**Published:** 2010-04-20

**Authors:** Vitaly Balan, Pratima Nangia-Makker, Avraham Raz

**Affiliations:** Karmanos Cancer Institute, Wayne State University, 110 E. Warren Avenue, Detroit, MI 48201, USA; E-Mails: vitaly.balan@gmail.com (V.B.); makkerp@karmanos.org (P.N.M.)

**Keywords:** galectins, biomarkers, cancer

## Abstract

Galectins are a group of proteins that bind β-galactosides through evolutionarily conserved sequence elements of the carbohydrate recognition domain (CRD). Proteins similar to galectins can be found in very primitive animals such as sponges. Each galectin has an individual carbohydrate binding preference and can be found in cytoplasm as well as in the nucleus. They also can be secreted through non-classical pathways and function extra-cellularly. Experimental and clinical data demonstrate a correlation between galectin expression and tumor progression and metastasis, and therefore, galectins have the potential to serve as reliable tumor markers. In this review, we describe the expression and role of galectins in different cancers and their clinical applications for diagnostic use.

## 1. Introduction

To date the galectin family includes a total of 15 proteins and is divided into three groups based on their protein structure. The prototype galectins, also called dimeric, contain one CRD (galectins-1,2,5,7,10,11,13,14,15), the tandem repeat galectins also called biCRD contains two CRDs (galectins-4,6,8,9,12) and the chimera type galectin consists of a large N-terminal region connected to a CRD (galectin-3).

Intensive studies demonstrate that galectins bind to other proteins through intracellular protein-protein interactions and lectin-carbohydrate interactions. Galectins function inside the cells in both carbohydrate dependent and independent manners and can regulate signal transduction as well as epithelial morphogenesis via an effect on centrosome biology [1]. Recently, galectins have been shown to bind glycans on the surface of potentially pathogenic microorganisms, and function as recognition and effector factors in innate immunity [2]. To date, a series of experimental and clinical evidences have been reported to support a correlation between galectin expressions and neoplastic transformation. Consequently, diagnostic and prognostic relevance of galectins have been shown, though there are still some conflicting data regarding some types of tumor tissues. The recent findings show that expression of galectins is elevated with neoplastic progression in certain malignancies, and therefore, galectins are expected to serve as reliable tumor markers. Despite the established role of galectins in numerous diseases, there is no clinically proven medication that targets these proteins, although a number of clinical trails were launched with its inhibitors. However, galectins play an important role as potential biomarkers that may help to identify the disease and serve as a therapeutic target. In this review we will summarize data on the role and expression of various galectins in different cancers with a focus on the probability of their use as cancer biomarkers. More citations have been made to galectin-3, since it is the most studied galectin. Galectin-3 is known for its role in tumorigenesis and progression through regulating cell proliferation, apoptosis, cell adhesion, invasion, angiogenesis and metastasis by binding to the N-acetyllactosamine moiety of cell surface glycoproteins or glycolipids [1,3,4]. Moreover, the presence of a collagen-like domain makes it susceptible to rapid and efficient cleavage by matrix metalloproteinases (MMP), in particular by MMP-2, MMP-9, and membrane type 1-MMP at the Ala^62^–Tyr^63^ peptide bond, resulting in the generation of a 22 kDa cleaved product [5]. Galectin-1 is a pleotropic homodimeric galectin that has been purified from numerous sources by lactose affinity chromatography. It is a 14 kDa polypeptide that assembles in homodimers and is almost exclusively composed of a single CRD. Galectin-4 was purified from intestine by affinity chromatography on lactose. This 36 kDa polypeptide is composed by two CRDs joined by a link peptide. Galectin-4 is synthesized as a cytosolic protein, but can be secreted similarly to other galectins. In cultured cells, intracellular galectin-4 may promote resistance to nutrient starvation, whereas as an extracellular protein it can mediate cell adhesion. Galectin-8 is a 35 kDa polypeptide composed of two CRDs joined by a link and was cloned from rat liver and from the human prostate carcinoma by the technique of surface epitope masking. Galectin-9 contains two homologous CRDs in a single polypeptide chain, separated by a linker. The N-terminal CRD of galectin-9 forms homodimers both in the crystal form and in solution, and the three-dimensional structure is different from the canonical twofold symmetric dimers seen for galectin-1 and -2 [6]. Galectin-9 is expressed by a variety of tumor cells and play an important role in tumor immunity by regulating the survival, proliferation and migration of both tumor cells themselves and immune cells in the tumor microenvironment [7].

## 2. Discussion

### 2.1. Galectins in Breast and Ovarian Cancer

Several early studies indicated a reduced expression of galectin-3 in advanced histological grades of breast cancer [8,9]. These findings are in conflict with *in vitro* studies involving MDA-MB-435 and BT-549 breast cancer cell lines in which a direct correlation between galectin-3 expression and metastatic and invasive potential of cells was observed [10,11]. Shekhar *et al.* indicated that the expression of galectin-3 mRNA and protein in human breast tumors and xenografts is not only associated with specific morphological precursor-subtypes of breast cancer but also demonstrate alterations in expression from luminal to peripheral (proximal to stroma) epithelial cells that is coincident with acquisition of invasive potential [12]. The role of galectin-3 in breast cancer progression was also demonstrated by using three-dimensional co-culture system that recapitulates *in vivo* reciprocal functional breast epithelial-endothelial cell-cell and cell-matrix interactions [13].

Tubular carcinomas (TC) are distinct low-grade breast neoplasms exhibiting homogeneous round-to-oval invasive tubular structures in more than 70% of tumoral glands, and distributed in fibrous stroma. TC is associated with a particularly favorable prognosis and a low rate of lymph node metastases. The metastatic potential of these lesions is extremely low if strict morphological criteria are applied, which explains their excellent prognosis, especially when tumor size is less than 2 cm (stage T1). Numerous reports have demonstrated a strong association between histological grade and survival in breast carcinomas [13]. However, G1 breast tumors are a heterogeneous group of lesions comprising various histological subtypes, such as TC, mucinous and cribriform carcinomas. It is difficult to distinguish between non-metastatic and metastatic carcinomas and decide about right treatment at very early stages. The higher expression of galectin-3 in TC and its focal staining (apical) pattern suggests that within the group of G1 carcinomas, galectin-3 expression varies according to histological type, and may correlate with prognosis and metastatic potential [14].

It was recently demonstrated that galectin-3 is a surrogate diagnostic marker for MMP activity in growing breast tumor. The gelatinases MMP-2 and MMP-9, which specifically degrade collagen IV, are important for initiation and development of tumor vascularization. Dependency of tumor angiogenesis on the activity of these MMPs renders this step a likely target of synthetic MMP inhibitors. Failed therapies directed against matrix metalloproteinases (MMP) in cancer patients may be attributed, in part, to lack of diagnostic tools to differentiate between pro-MMPs and active MMPs, which indicate whether a treatment is efficacious or not. Because galectin-3 is cleavable by MMPs and is co-localized with active MMPs, antibodies recognizing cleaved and non-cleaved forms of galectin-3 can indicate the presence of active MMPs in a cancer. Galectin-3 cleavage is an active process during tumor progression and can be used as a simple, rapid, and reliable surrogate marker for MMPs activity [15]. As the cleaved protein increases during cancer progression, the levels of intact protein decrease and thus explain the discrepancy in the previous data emphasizing the usefulness of galectin-3 as a breast cancer biomarker.

Galectin-9 contributes to the aggregation of breast cancer cells and the relation between galectin-9 expression in tumor tissue and distant metastasis in patients with breast cancer was demonstrated. In a research conducted by Irie *et al.* patients with breast cancer were tested for galectin-9 expression and followed up for 14 years. Tumors from 50% of the patients were positive for galectin-9. However 90% of the patients with distant metastasis were galectin-9 negative. This data suggest that galectin-9 is a possible prognostic factor with anti-metastatic potential in breast cancer [16].

Tissues from reduction mammoplasties without cytopathological abnormalities and normal tissues surrounding the malignant component showed minimal or no galectin-4 expression in most cases [17]. Huflejt *et al.* demonstrated that a weak induction of galectin-4 was associated with epithelial hyper-proliferation, and the atypical component in benign breast biopsies often showed very high intracellular expression. The highest levels of galectin-4 expression were found in the ductal carcinoma *in situ* cases and in a subset of infiltrating ductal carcinomas [17]. Because of distinct induction of galectin-4 in breast cancers, it may be a valuable diagnostic marker and target for the development of inhibitory carbohydrate-based drug.

Galectins are also over-expressed in ovarian cancers. To find if galectin-3 can be used as marker in ovarian cancer, urinary galectin-3 levels to stage cancer disease was examined. Determination of galectin-3 levels in urine of cancer patients and healthy controls established a strong correlation between the stages of the disease with galectin-3 concentration. Although only 16 patients with ovarian cancer were included in the study the significant statistical differences in galectin-3 concentration between normal and cancer patients was observed [18].

Mucinous epithelial ovarian cancers (MOC) are clinically and morphologically distinct from the other histological subtypes of ovarian cancer. The results published by Heinzelmann-Schwarz *et al.* show that MOC express a genetic profile that both differs and overlaps with other subtypes of epithelial ovarian cancer. Concordant with its histological phenotype, MOC express genes characteristic of mucinous carcinomas of varying epithelial origin, including intestinal carcinomas. Differences in gene expression between MOC and other histological subtypes of ovarian cancer were confirmed by qRT-PCR and/or immunohistochemistry. In particular, galectin-4 was highly and specifically expressed in MOC, but expressed at lower levels in benign mucinous cysts and borderline (atypical proliferative) tumors, supporting a malignant progression model of MOC [19]. Hence galectin-4 may have application as an early and differential diagnostic marker of MOC. 

Investigation of the expression of epidermal growth factor receptor (EGFR), galectin-3 and cyclin D1 in a cohort of ovarian serous carcinomas with regard to outcome and clinicopathologic parameters shows that galectin-3 and cyclin D1 immunostaining decreased from serous cystadenomas and serous borderline ovarian tumors to the carcinomas significantly. Galectin-3 immunostaining of any pattern was not related to grade or stage in cancers; mere cytoplasmic expression was associated with poor outcome. This study indicates that with regard to EGFR and cytoplasmic galectin-3 immunoexpression, multiple marker testing may be an adjunct in the identification of high-risk ovarian serous cancers [20]. Taken together these data support the usefulness of galectins as biomarkers in both breast and ovarian cancer. However, we think that the most significant clinically relevant function of galectin-3 is as a marker that can distinguish tubular carcinoma from mucinous and cribriform carcinomas. Galectin-3 can be a tool to distinguish the proactive and active forms of MMP-2 and -9 and galectin-4 as a marker of MOC in ovarian cancer. 

### 2.2. Galectins in Bladder Cancer

The expression of galectin-1 and galectin-3 was investigated in 38 human bladder transitional cell carcinomas of different histological grade and clinical stage and in five normal urothelium samples. Galectin-1 mRNA levels were highly increased in most high-grade tumors compared with normal bladder or low-grade tumors. Western blot and immunohistochemical analysis of normal and neoplastic tissues revealed a higher content of galectin-1 in tumors. Galectin-3 mRNA levels were also increased in most tumors compared with normal urothelium, but levels were comparable among tumors of different histological grade [21].

Data obtained by Sakaki *et al.* demonstrate that serum levels of galectin-3 in patients with bladder cancer are significantly higher than the control group. Median value of serum galectin-3 concentration was 1068 pg/mL in the cancer group *vs.* 584 pg/mL in controls [22]. A standard method for diagnosis of bladder cancer is urine cytology and cystoscopy. However, cystoscopy is invasive and expensive and specificity of urinary markers decreases in cases with pyuria in cystitis and urolithiasis. Contrary to urinary markers, serum level of galectin-3 is not affected by pyuria. For that reason the measurement of serum galectin-3 will be helpful in diagnosis of bladder cancer, if it is clinically suspected.

### 2.3. Galectins in Gastric and Colon Cancer

Lotan *et al.* first demonstrated that galectin-3 expression was different in gastric carcinomas compared to normal tissue [23]. The galectin-3 level was significantly higher in the primary tumor compared to adjacent normal tissue in 55% of the well-differentiated tubular carcinoma cases and in 50% of stage III and IV tumors. Galectin-3 was detected in normal gastric epithelial cells and in all gastric carcinoma specimens, albeit in varying amounts. Galectin-3 expression in liver metastases from well-differentiated tubular primary gastric carcinomas was higher in 31% of the cases relative to the corresponding primary cancers and expression in metastases from poorly differentiated gastric carcinomas in lymph nodes was higher in 38% of the cases compared to the primary cancers [23].

These data were confirmed by Baldus *et al.*, who showed that galectin-3 expression was higher in primary gastric adenocarcinomas compared to the normal tissues. However, there was no correlation between membrane-bound *versus* cytoplasmic galectin-3 with histopathological differentiation parameters (according to the WHO and Laurén classifications) or tumor progression. Nuclear galectin-3 reactivity was significantly stronger in diffuse-type cancers compared to the intestinal-type tumors [24]. The data obtained during this study demonstrated that a prognostic value of galectin-3 regarding patient survival could not be established. 

In another study, positive galectin-3 expression was observed in 84% of the gastric cancer cases and galectin-3 expression in gastric carcinoma compared with that in gastric tissues adjacent to the cancers demonstrated a significant increase [25]. However, a significantly stronger expression of galectin-3 in cancer tissues was observed only in papillary and poorly differentiated adenocarcinoma. A significant correlation of galectin-3 expression was observed with tumor progression in overall cases, and in poorly differentiated adenocarcinoma the expression of galectin-3 in metastatic lymph nodes was stronger than the primary cancer [25]. In another study, a negative correlation was found between the expression levels of Li-cadherin and Galectin-3 in gastric cancer, while galectin-3 expression was related to TNM staging (Tumor, Nodes, Metastases). Malignant gastric tissues expressed high levels of galectin-3 and fascin-1, compared with normal gastric tissues. Silencing of galectin-3 resulted in altered cancer cell morphology, reduced fascin-1 expression, decreased cell motility, and reduced malignant cell invasion. Galectin-3 over-expression reversed these effects [26].

Hippo *et al.* performed global analysis on differential gene expression of a scirrhous gastric cancer cell line (OCUM-2M) and its derivative sublines with high potential for metastasis to the peritoneal cavity (OCUM-2MD3) and lymph nodes (OCUM-2MLN). The analysis revealed two functional gene clusters with altered expression: down-regulation of a cluster of squamous cell differentiation marker genes such as small proline-rich proteins and up-regulation of a cluster of antigen-presenting genes. Following analysis of six gastric cancer cell lines by Northern blot demonstrated preferential up-regulation of galectin-4 and few other proteins only in cells with a high potential for peritoneal dissemination and a low expression level in all of the cells with a low potential for peritoneal dissemination [27].

Based on these data galectin-3 and probably galectin-4 might be useful tumor markers for gastric cancers with respect to tumor progression and potentiality of lymph node metastasis especially in certain histological types of gastric cancer.

The results published regarding expression of galectin-3 in colon cancer are conflicting. While most of the publications reported increased expression [28,29,30,31,32,33], some demonstrated decreased expression of galectin-3 in colon cancer [34,35] compared to the normal tissue. In none of the studies however, were galectin-3 levels reported to stay unchanged in normal *versus* tumor tissue.

A similar study demonstrate that not only galectin-3 but also galectin-1 expression levels correlate with the degree of dysplasia, suggesting that galectin-1 is related to malignant progression, while galectin-8 has been associated with suppressor activity in colon cancer [30].

The paper published by Nagy *et al.* indicates that the galectins -1,3,4 and -8 are associated with significant and separate prognostic values that depend on the Dukes stage of the colon tumor. These authors observed a significant prognostic value associated with galectins-1,3, and -4 in Dukes A and B colon tumors and galectin-8 in Dukes C and D [36]. The same feature was observed when galectin-4 and galectin-8 were analyzed in the complete series. A marked decrease in immunohistochemical expression of galectin-8 occurred with malignancy development in human colon tissue. The more aggressive the tumor, the less galectin-8 it harbored [36]. 

While galectin-3 demonstrates consistent results as biomarker in gastric cancer, additional research is required to confirm its efficacy as marker in colon cancer. 

### 2.4. Galectins in Melanomas

In series of papers Vereecken *et al.* showed a correlation between galectin-3 expression and malignant potential in primary melanoma lesions. To study the possible correlation between galectin-3 expression and the malignant potential in primary melanoma lesions they conducted an immunohistochemical study with monoclonal anti-galectin-3 antibody in a series of primary and metastatic melanoma lesions as well as benign skin pigmented lesions. The expression of galectin-3 was higher in thin primary melanoma lesions than in benign pigmented skin lesions or metastases and seemed to correlate inversely with the aggressiveness as estimated by the Breslow index, which is recognized as the main prognostic factor in melanoma [37]. To further validate galectin-3 as prognostic or diagnostic marker they determined whether an increase in serum galectin-3 production could be found in patients with advanced metastatic melanoma. Galectin-3 concentration was shown to be significantly higher in the group of patients with melanoma compared with healthy volunteers, and galectin-3 concentration significantly correlated with both serum lactate dehydrogenase and C-reactive protein in the melanoma group [38]. The recent study of this group clearly demonstrates that galectin-3 could be of prognostic value in melanoma patients; more precisely, this protein has a strong independent prognostic signification with a cut-off value of 10 ng/mL [39]. Other groups also confirm these findings and furthermore show that melanocytes accumulate galectin-3 with tumor progression, particularly in the nucleus. The strong association of cytoplasmic and nuclear expression in lesions of sun-exposed areas suggests an involvement of UV light in activation of galectin-3 [40]. 

### 2.5. Galectins in Prostate Cancer

The first reports about expression galectin-1 and -3 in prostate cancers showed that galectin-1 was expressed in most cases of all four histologic types. In contrast, galectin-3 expression was significantly decreased in primary carcinoma and metastatic disease compared with normal and premalignant tissue. Galectin-3 expression in primary tumors tended to be less than that of surrounding normal glands [41,42]. Another report using 148 human primary prostate carcinoma samples confirmed that galectin-1 accumulated in the stroma and associated fibroblasts but was not detected in normal, intraepithelial neoplasia or carcinoma cells [43]. The association between over-expression of galectin-1 in the stroma of the malignant tissue and aggressiveness of the tumor emphasizes a significant role for galectin-1 in the acquisition of the invasive phenotype [43]. The expression of galectin-3 was also examined in 145 prostate carcinoma samples using immunohistochemistry [44]. Most of the non-tumoral prostatic glands exhibited moderate immunostaining for galectin-3 localized both in nucleus and cytoplasm. In prostatic cancer cells, galectin-3 was usually not expressed or decreased compared with the normal glands. Interestingly, when galectin-3 was detected in the cancer cells, it was consistently excluded from the nucleus and only present in the cytoplasmic compartment [44]. 

Ahmed *et al.* demonstrated that in prostate cancer cells as well as in tissues, galectin-3 promoter was highly methylated in human prostate cancer tissue but not in normal tissue. The authors suggest that the methylation of galectin-3 promoter may constitute a powerful tool for early diagnosis of prostate cancer [45].

A report recently published by our group revealed the role of galectin-3 in progression of prostate cancer. Immunohistochemical analysis of a prostate tissue array using differential galectin-3 staining demonstrated an increased cleavage of galectin-3 during the progression of prostate cancer. Galectin-3 knockdown in prostate cancer cells by small interfering RNA was associated with reduced cell migration, invasion, cell proliferation, anchorage-independent colony formation, and tumor growth in nude mice. The data implicate galectin-3 in prostate cancer progression and suggest that galectin-3 may serve as both a diagnostic marker and therapeutic target for future disease treatments [46].

### 2.6. Galectins in Lung Cancer

Yoshimura *et al.* investigated mRNA levels of galectin-3 in lung cancer cell lines and in tumor tissue obtained by thoracoscopic biopsy using qRT-PCR. A population of the non-small-cell lung cancers examined was found to over-express the galectin-3 gene at levels three times higher than those of normal epithelial cells. In contrast, all small-cell lung cancers either failed to express the gene or expressed it at a very low level. These results, suggest that galectin-3 may play a role in the process of metastasis in non-small-cell lung cancer that over-expresses galectin-3, but not in small-cell lung cancer. The authors concluded that galectin-3 may be a phenotypic marker that excludes small-cell lung cancer and may represent a novel target molecule in non-small-cell lung cancer therapy [47]. 

These findings were confirmed by other groups [48,49,50,51]. Immunohistochemical expression of galectin-3 was evaluated in a consecutive series of 81 radically resected non-small cell lung carcinomas (NSCLCs). It was reported that the main pattern of galectin-3 expression was cytoplasmic. The authors also demonstrated that concomitant expression of nuclear galectin-3 and TTF-1 was independently associated with a worse clinical outcome [52]. Compared with healthy individuals, serum levels of galectin-3 in patients with lung cancer were significantly elevated [53]. All together these data suggest that galectin-3 can be a reliable marker for lung cancer.

Some studies showed that the lung cancer could also be diagnosed by quantifying the galectin-8 expression level. The presence of galectin-8 in lung tumor cells and its absence or very low levels in normal lung tissues permits the use of monoclonal antibodies (Po66) for the prevention and treatment of lung cancer. Prior to the knowledge that this antibody was able to specifically bind galectin-8, it was used to detect human lung squamous cell carcinoma by immunoscintigraphy [54,55]. This clinical investigation showed that Po66 antibody was able to detect specifically the lung squamous cell carcinoma recurrence [55]. 

### 2.7. Galectins in Lymphoma

Although galectin-3 is lowly expressed in normal lymphocytes and not detected in a number of lymphoma cell lines, its expression is markedly up-regulated in some lymphoma cell lines. Among a variety of lymphomas, anaplastic large-cell lymphoma (ALCL) neoplastic cells appear to be the only ones that consistently express galectin-3. Immunohistochemical detection of this lectin, therefore appears to be useful for identification of this subtype of lymphoma [56]. Recently, the role of galectin-3 in the prognosis and extranodal involvement of diffuse large B-cell lymphomas (DLBCL) was demonstrated. Galectin-3 expression has never been associated with prognosis in non-Hodgkin’s lymphomas. However, high galectin-3 expression was found in the majority of DLBCL samples and cell lines, suggesting that galectin-3 may be associated with aggressive lymphoma subtypes [57]. 

### 2.8. Galectins in Gliomas and Meningiomas

Initial immunohistochemical studies reported conflicting results on the expression of galectin-3 in human gliomas. An initial study found a positive correlation with the WHO-grade [58], while an inverse correlation was reported later [59]. In 2001 Strik *et al.* tried to clarify this issue and studied cellular and regional distribution of galectin-3 and compared it with immunoreactivity to the macrophage marker CD68 in 53 gliomas (WHO-grades II–IV) by immunohistochemistry. The expression of galectin-3 showed a marked variability, however the positivity for the macrophage/microglial marker CD68 was significantly correlated with positivity for galectin-3. They concluded that the main source of galectin-3 was tumor-infiltrating macrophages, which cannot always be clearly distinguished from glioma cells by pure morphological criteria and the marked variability of galectin-3 positivity in the different WHO-grades prevents a diagnostic use for the grading of gliomas [60]. 

Meningiomas, derived from the meninges are unique tumors with mesenchymal and epithelial differentiating potentials. Das *et al.* demonstrated galectin-3 expression in 100% of samples studied, while 57% showed detectable levels of galectin-3 in the nucleus [61]. In the latest study researchers investigated galectin-3 expression in 409 cases of surgically resected primary brain tumors, including various glio-neuronal tumors, pituitary adenomas, meningiomas and Schwannomas. In normal brain tissues, galectin-3 was robustly expressed in normal astrocytes, endothelial cells and macrophages. It showed consistent and diffuse positivity in 100% of the pilocytic astrocytomas, pleomorphic xanthoastrocytomas (PXA), Schwannomas, meningiomas, capillary hemangioblastomas, as well as in ependymomas, but it was completely negative in the diffuse astrocytomas, anaplastic astrocytomas, both low- and high-grades of the oligodendrogliomas, central neurocytomas, and medulloblastomas. Definitely positive but heterogeneous expression was found in various tumors including subependymal giant cell astrocytomas (SEGA), classic glioblastoma multiforme, anaplastic oligoastrocytomas, CNS primitive neuroectodermal tumors (CNS PNETs), and hemangiopericytomas. Based on this data the authors conclude that galectin-3 is differentially expressed in various brain tumors, and thereby, is a helpful biomarker in making differential diagnoses, especially in cases where a morphological diagnosis is controversial [62].

### 2.9. Galectins in Pancreatic Cancer

Galectin-3 is heavily expressed in cytoplasmic and nuclear regions of 50% of normal human pancreatic tissue. However comparison between expression of galectin-3 in ductal cells in chronic pancreatitis and cancerous pancreatic tissue demonstrates that its expression was increased over normal tissue and was more uniform [63]. Normal hamster pancreatic ducts showed weak or no expression of galectin-3, while hyperplastic pancreatic ductal cells from BOP-treated hamsters heavily expressed galectin-3. Galectin-3 expression in ductal cells in cancerous pancreatic lesions was also significantly increased [63]. 

Berberat *et al.* studied expression of galectin-1 and galectin-3 in tissue samples of 33 primary pancreatic cancers and in tumor metastases in comparison to 28 normal pancreatic samples. Northern blotting and Western blotting analysis showed significantly higher galectin-1 and galectin-3 mRNA and protein levels in pancreatic cancer samples compared to normal controls. No relationship between the galectin-1 and galectin-3 mRNA levels and the tumor stage or between the immunohistochemistry staining score and the tumor stage was found. However, galectin-1 mRNA levels and the immunohistochemistry staining score were significantly higher in poorly differentiated tumors compared with well/moderately differentiated tumors, whereas for galectin 3 no differences were found [64]. 

In another study decreased expression of galectin-3 was associated with advanced stage, tumor de-differentiation, and metastasis in ductal adenocarcinoma of the pancreas. Authors concluded that galectin-3 expression might be a useful prognostic marker for survival in ductal adenocarcinoma of the pancreas [65]. The proteomic analysis of pancreatic ductal adenocarcinoma compared to normal tissue demonstrates that galectin-1 expression was highly correlated to tumor histology and stage [66].

Taken together the expression pattern of galectin-1 and galectin-3 in pancreatic cancer tissues indicates that these proteins can be useful biomarkers in this type of cancer. Especially important is the expression of galectin-1, which can help to distinguish between poorly differentiated tumors and well/moderately differentiated tumors.

### 2.10. Galectins in Squamous Cancer

Initial evaluation of the expression of galectins-1 and -3 in head and neck squamous cell carcinoma (HNSCC) was performed in fourteen HNSCC cell lines and four primary tumor specimens. The expression pattern of galectins appears to be associated with degree of squamous differentiation, suggesting a potential role for galectins that were localized to the cell surface [67].

Later investigations showed that galectin-3 expression was positively associated with tumor keratinization and histologic grade. A significant correlation was found between galectin-3 tumor positivity and longer relapse-free and overall survival. In univariate analysis, high-grade (grade 3 or 4) tumors, nonkeratinizing tumors, and galectin-3-negative tumors showed a significantly increased risk of relapse and death. In multivariate analysis, only galectin-3 expression retained an independent prognostic significance for both relapse-free and overall survival. Taken together these data suggest that the absence of galectin-3 expression is an independent negative prognostic marker in laryngeal SCC patients [68].

Gabius and co-workers demonstrated that galectin-3 binding might be used as a prognostic marker for squamous carcinoma. The results obtained from analysis of data from patients suffering from advanced squamous carcinoma of the larynx and oropharynx revealed that galectin-3 expression in tumor cells is positively and significantly related to prognosis [69]. The application of the endogenous galectin-3 as a histochemical marker defines a new prognostic tool in patients with laryngeal and oropharyngeal squamous cell carcinoma. It was also illustrated that expression of galectin-3 reactive glycoligands is differentiation-dependent in normal as well as malignant squamous cells [69,70,71]. The level of galectin-3 expression in relation to neoplastic progression of hypopharyngeal squamous cell carcinomas (HSCCs) and laryngeal squamous cell carcinomas (LSCCs) demonstrate an association between the level of galectin-3 expression and neoplastic progression in these carcinomas [72].

Not only galectin-3 can be used as a marker in squamous cell carcinomas, expression of galectin-7 seems to be significantly reduced in malignant cells of squamous epithelia of both ectodermal and endodermal origin, thus allowing use of galectin-7 as differentiation marker of epithelial malignancies [73]. 

HNSCCs display significant levels of galectin-7 by immunohistochemical assays, but this galectin cannot be detected in the blood of HNSCC patients, while galectin-3 and -1 levels differed significantly in serum of healthy volunteers and HNSCC patients. Galectin-3 concentrations in sera from the patients with a metastatic disease were significantly higher than in sera from the patients with localized tumors. The determination of circulating levels of galectin-1 and -3 could be used to monitor the progression of their disease or their response to therapy [74].

### 2.11. Galectins in Thyroid Cancer

In 1995 Xu *et al.* found that all thyroid malignancies of epithelial origin and a metastatic lymph node from a papillary carcinoma expressed high levels of both galectin-1 and -3. In contrast, neither benign thyroid adenomas nor adjacent normal thyroid tissue expressed galectin-1 or galectin-3. Although these results were based only on 32 specimens from thyroid malignancies and 33 specimens from adjacent normal thyroid tissue, they suggest that galectin-1 and galectin-3 may be associated with malignant transformation of thyroid epithelium and may potentially serve as markers for distinguishing benign thyroid adenomas from differentiated thyroid carcinomas [75]. Following investigations clearly show that galectin-3 expression in paraffin-embedded cytological thyroid sediments (cell blocks) obtained by fine-needle aspiration biopsy was significantly higher compared to their histological counterparts. The sensitivity and specificity of galectin-3 immunodetection alone in discriminating benign from malignant thyroid lesions were more than 99% and 98% respectively, and the positive predictive value and diagnostic accuracy were 92% and 99% [76,77,78,79,80,81]. Taken together these data suggested that cytoplasmic galectin-3 staining could be a reliable, easy, and cheap marker for presurgical diagnosis of follicular carcinomas and an even more suitable one for papillary carcinomas. However some researchers raise a question regarding ability of galectin-3 to serve as universal marker. They found that galectin-3 expression is also present in Hashimoto thyroiditis and this reveals some limitations in nodule or multiple nodules of benign character [82]. However if the diagnosis of Hashimoto thyroiditis is excluded, then the usefulness of the galectin-3 in the diagnosis of malignancy still remain very high [82]. Other researchers also found that diagnostic problems may arise in the presence of Hurthle cell proliferation or minimally invasive follicular carcinoma [83].

Mehrotra *et al.* found that galectin-3 expression was observed in majority of the carcinomas. However, he also demonstrated that a large proportion of follicular adenomas (72%) and multinodular goitres (57%) also expressed galectin-3 and its expression was no greater in follicular carcinomas than in follicular adenomas. Based on these data he concluded that galectin-3 is not a reliable immunohistochemical marker to distinguish benign from malignant thyroid follicular lesions [84]. Some other researchers confirm these findings and conclude that galectin-3 cannot reliably distinguish malignant and benign lesions [85]. However, most recent investigations in this area did not confirm these findings and support usefulness of galectin-3 as biomarker for thyroid cancer [86,87,88,89]. For example, recently Bartolazzi and colleagues by using standardized method of galectin-3 staining on FNA-derived cell blocks demonstrated that 381 (88%) out of 465 samples of the follicular thyroid nodules referred for surgery were classified correctly pre-operatively [90]. These findings were confirmed by Wiseman *et al.*, who demonstrated that most significant markers for differentiated thyroid cancer diagnosis were galectin-3 together with some other proteins [91]. This issue was also discussed in very valuable review by Griffith *et al.* and with overall conclusion confirming the worthiness of galectin-3 as biomarker in thyroid cancer [92]. 

## 3. Concluding Remarks

**Figure 1:**
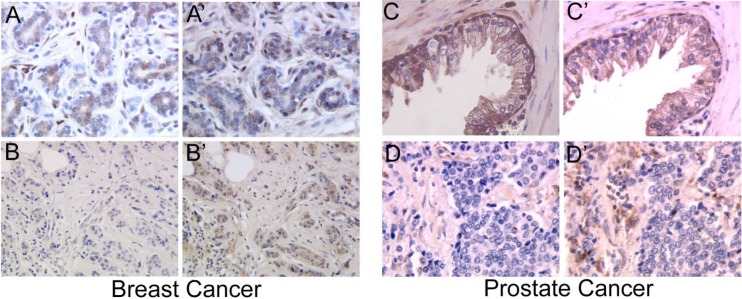
Galectin-3 cleavage in breast cancer and prostate cancer: Representative sections showing differential immunohistochemistry of breast and prostate cancer tissue array with anti-galectin-3 monoclonal (staining intact protein) and polyclonal (staining intact and cleaved protein) antibodies. A–D: intact protein; A’–D’: intact + cleaved protein. A–A’: Normal breast ducts; B–B’: infilterating ductal carcinoma of the breast; C–C’: Normal prostate ducts; D–D’: prostate metastasis. Brown color represents positive staining. Note similar staining patterns in normal ducts using both the antibodies, while polyclonal antibody stained a lot more cells in carcinoma and metastasis indicating galectin-3 cleavage. 400×.

Based on above data we can conclude that galectins expression is modulated by several oncogenic stimuli and shown to be up or down-regulated in different human tumors. However the disparity in expression level was also observed in the same tumor type. The reason for this disparity can be different antibodies used by various research groups. It has been demonstrated, for example that a rat monoclonal antibody (TIB166) can recognize intact galectin-3, while a polyclonal antibody developed against the whole molecule can recognize both cleaved and intact protein [15]. Using tissue array of human breast and prostate cancers it has been showed that galectin-3 is cleaved during cancer progression (Figure 1). The cleavage is also dependent on the presence of a single nucleotide polymorphism (rs4644) in galectin-3 gene, which results in the expression of MMP-2/-9 cleavable (H^64^) or non-cleavable (P^64^) galectin-3. This SNP has been found in 5 and 12% of Asian and Caucasian population respectively and increases to 82 and 37% respectively in breast cancer patients in these two races [93]. Taking this into consideration, the interpretation of the data can vary depending on whether galectin-3 monoclonal or polyclonal antibody was used, how many of the total analyzed samples expressed H^64^ galectin-3, whether the samples contained any active MMP-2 or -9.

In recent years, more emphasis has been given to characterization of the human tumors in order to develop individualized therapeutic regimen for patients. However, the tools that allow us to determine the risk factors responsible for the development and progression of cancer, and a patient’s response to particular targeted therapies are still under development. Some biomarkers are already being used as clinical tools, but the field is still in juvenile stage. More biomarkers need to be developed and their prognostic roles need to be developed in cohort with already developed biomarkers. Galectins, which are broadly expressed and evolutionarily conserved animal lectins, can play crucial biological roles in tumor cell-cell or cell-matrix interactions through their binding activities to the tumor cell surface carbohydrate ligands. Some of the galectins have also been shown to affect tumor cell survival, signal transduction, and proliferation mainly by interactions inside the cell. The differential expression levels of galectins in normal *versus* tumor tissue open the possibility of using these proteins as biomarkers to monitor cancer progression and determine clinical outcomes in patients. They also can be helpful in development of clinical tools to determine which patients are most likely to benefit from the therapy. For example, cleavage of galectin-3 by MMPs: galectin-3 can be used as a diagnostic marker to check the activity of MMPs during treatment with MMPs inhibitor. However, the data available today allow us to conclude that no one marker, including galectins, has proven to be a panacea and panel of markers should be used for accurate diagnosis. 
